# Evaluation of the effect of filtered ultrafine particulate matter on bleomycin-induced lung fibrosis in a rat model using computed tomography, histopathologic analysis, and RNA sequencing

**DOI:** 10.1038/s41598-021-02140-2

**Published:** 2021-11-22

**Authors:** Cherry Kim, Sang Hoon Jeong, Jaeyoung Kim, Ja Young Kang, Yoon Jeong Nam, Ariunaa Togloom, Jaehyung Cha, Ki Yeol Lee, Chang Hyun Lee, Eun-Kee Park, Ju-Han Lee

**Affiliations:** 1 Department of Radiology, Ansan Hospital, Korea University College of Medicine, 123, Jeokgeum-ro, Danwon-gu, Ansan-si, Gyeonggi, 15355 South Korea; 2 Medical Science Research Center, Ansan Hospital, Korea University College of Medicine, 123, Jeokgeum-ro, Danwon-gu, Ansan-si, Gyeonggi, 15355 South Korea; 3 Department of Radiology, College of Medicine, Seoul National University, Seoul National University Hospital, Seoul, 03080 South Korea; 4 Department of Medical Humanities and Social Medicine, College of Medicine, Kosin University, Busan, 49267 South Korea; 5 Department of Pathology, Ansan Hospital, Korea University College of Medicine, 123, Jeokgeum-ro, Danwon-gu, Ansan-si, Gyeonggi, 15355 South Korea

**Keywords:** Diseases, Medical research

## Abstract

We aimed to investigate the effect of chronic particulate matter (PM) exposure on bleomycin-induced lung fibrosis in a rat model using chest CT, histopathologic evaluation, and RNA-sequencing. A bleomycin solution was intratracheally administrated to 20 male rats. For chronic PM exposure, after four weeks of bleomycin treatment to induce lung fibrosis, PM suspension (experimental group) or normal saline (control group) was intratracheally administrated for 10 weeks. Chest CT was carried out in all rats, and then both lungs were extracted for histopathologic evaluation. One lobe from three rats in each group underwent RNA sequencing, and one lobe from five rats in each group was evaluated by western blotting. Inflammation and fibrosis scores in both chest CT and pathologic analysis were significantly more aggravated in rats with chronic PM exposure than in the control group. Several genes associated with inflammation and immunity were also upregulated with chronic PM exposure. Our study revealed that chronic PM exposure in a bleomycin-induced lung fibrosis rat model aggravated pulmonary fibrosis and inflammation, proven by chest CT, pathologic analysis, and RNA sequencing.

## Introduction

Exposure to ambient particulate matter (PM) is a major global health concern that leads to respiratory and cardiovascular mortality. A recent Global Exposure Mortality Model, based on 41 cohort studies in 16 countries, found that solid particles or liquid droplets suspended in the air are associated with almost 8.9 million deaths per annum worldwide^1,2^. In addition, many animal experiments have shown that adverse respiratory outcomes are associated with PM exposure, including exacerbations of idiopathic pulmonary fibrosis (IPF), chronic obstructive pulmonary disease (COPD), and asthma^3–10^, which highlight the molecular mechanism of PM’s induction of respiratory diseases. In addition, high concentrations of PM in a particular season or year-round have persisted in several countries over several years for various reasons. As with most environmental problems, this phenomenon caused by air pollution is not expected to be easily improved, so the effect of chronic exposure to PM on a diseased lung is an important research topic requiring evaluation.

Chest computed tomography (CT), a superior imaging method for diagnosing respiratory diseases in humans, can easily diagnose lung fibrosis without pathologic confirmation^11^. CT also provides an overview of all lung parenchyma and facilitates a gross assessment of the overall lung pathology. While some lung tissue not provided as slides may be excluded from pathologic analysis, it can be included in CT evaluation. Therefore, CT allows for an enhanced evaluation of the extent of fibrosis. Recent research evaluated polyhexamethylene guanidine (PHMG)‑induced lung injuries by chest CT^12,13^. In these studies, CT findings of lung inflammation and fibrosis due to PHMG were well correlated with pathological findings, proving the usefulness of chest CT in the evaluation of lung lesions in a rat model. However, there have been few papers verifying the pulmonary effects of PM using chest CT in both experimental animals and humans.

RNA-sequencing is a precise and sensitive tool for measuring global gene expression profiles. Recently, this technique has been used to investigate the mechanism of PM-induced cytotoxicity in cell and animal models^14–16^. It is important to clarify the global gene expression profiles in the lungs of PM-exposed rats using RNA-sequencing to better understand the harmful effects of PM on bleomycin-induced lung fibrosis.

Our study aimed to investigate the effects of chronic PM exposure on a bleomycin-induced lung fibrosis rat model using chest CT, histopathological evaluation, and RNA-sequencing.

## Results

### Quality analysis of CT images

Among the 20 rats included in this experiment, CT images of 17 rats had unimpaired detectability with high diagnosis confidence, whereas one rat in the experimental group and two rats in the control group had mild but insignificant detectability impairment. There was no significant difference in the image quality score in both groups (4.90 ± 0.32 vs. 4.80 ± 0.42, P = 0.542).

### Evaluation of CT findings

**Figure 1:**
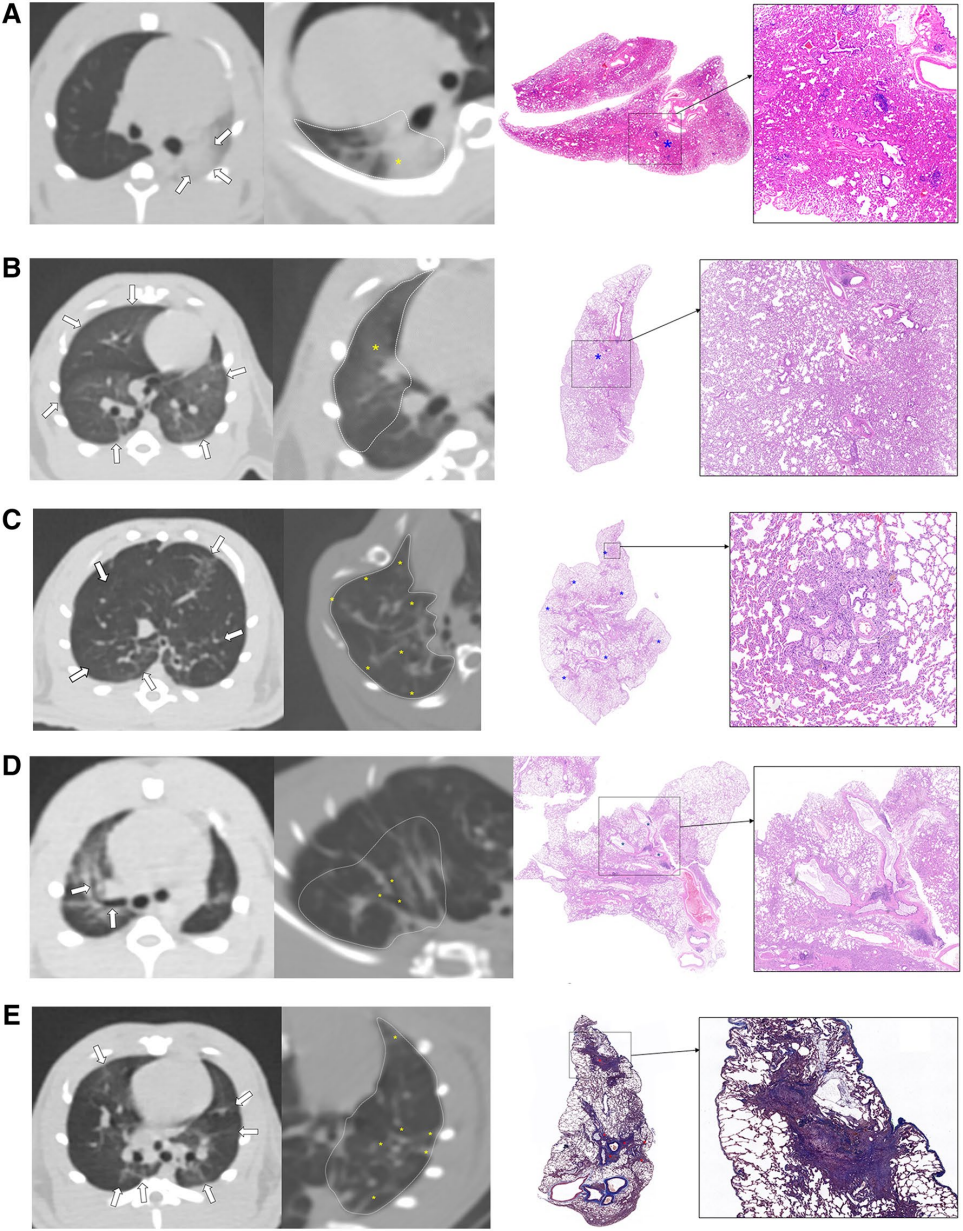
Examples of inflammation and fibrosis in chest CT imaging and correlated histologic findings. (**A**) Consolidation is shown in the left lower region (white arrows). This consolidation in the differently reformatted CT image of the same rat and same lung region (yellow asterisk) is correlated with the histology section (blue asterisk, hematoxylin and eosin stain [H&E], X2). Histologic finding shows widening of interalveolar spaces with congestion. Several foci of lymphoid aggregates are also seen (box, H&E stain, X40). (**B**) Ground glass opacities (GGO) are shown in both lungs (white arrows). This GGO in a differently reformatted CT image of the same rat (yellow asterisk) is correlated with the histology section (blue asterisk, H&E stain, × 2). The histologic finding shows mild thickening of the alveolar septa with some histiocytes in the alveoli (in box, H&E stain, X20). (**C**) Centrilobular nodules are shown in both lungs (white arrows). Centrilobular nodules in differently reformatted CT image of the same rat (yellow asterisks) are correlated with the histology section (blue asterisks, H&E stain, X2). The histologic finding shows peribronchiolar inflammatory cell infiltration with thickening of the alveolar septa. Foamy histiocytes and pigmented macrophages are also seen in the alveoli (box, H&E stain, X100). (**D**) Bronchiectasis is in the right superior lobe (white arrows). Bronchiectasis in a differently reformatted CT image of the same rat in the right inferior lobe (yellow asterisks) is correlated with the histology section (blue asterisks, H&E stain, X2). The histologic finding shows cystic dilatation of bronchus with mucus secretion in the bronchus (box, H&E stain, X20). and (**E**) Multifocal linear densities in both lungs (white arrows). Multifocal linear densities in a differently reformatted CT image of the same rat in the left lower region (yellow asterisks) are correlated with the histology section (red asterisks, Masson's trichrome [MT] stain), X2). The histologic finding shows a confluent fibrotic band with obliteration of the alveoli (box, MT stain, X50).

The results of the CT imaging analysis are shown in Table 1 and examples of the CT findings are shown in Fig. 1. Detection of consolidation and bronchiectasis/linear densities was significantly greater in the experimental group than in the control group (P = 0.011 and P = 0.033, respectively). There was no consolidation in the control group, whereas 60% of the experimental group demonstrated consolidation. Bronchiectasis suggestive of lung fibrosis was detected in all rats (100%) and 50% of the control group.

**Table 1:** The CT score findings in the experimental and control groups.

	Experimental group	Control group	P-value
**Presence of each CT finding, n (%)**			
Consolidation	6 (60%)	0	0.011
GGO	10 (100%)	10 (100%)	N/A
Centrilobular nodules	10 (100%)	8 (80%)	0.474
Bronchiectasis/linear densities	10 (100%)	5 (50%)	0.033
**CT finding scores**			
Consolidation score	1.10 ± 0.99	0	0.023
GGO score	3.60 ± 0.84	1.20 ± 0.42	< 0.001
Centrilobular nodules score	3.00 ± 0.00	0.80 ± 0.42	< 0.001
Bronchiectasis/linear densities score	3.80 ± 0.42	1.10 ± 1.37	< 0.001

Note – GGO, ground-glass opacity.

The consolidation score, GGO score, centrilobular nodules score, and bronchiectasis/linear densities score were significantly higher in the experimental group than in the control group (all P < 0.05).

### CT evaluation of inflammation and fibrosis

Table 2 shows the comparison for inflammation and fibrosis in chest CT between the experimental and control groups. The total CT score was significantly higher in the experimental group than in the control group (21.10 ± 6.49 vs. 6.60 ± 1.58, P < 0.001). CT scores were significantly higher in the experimental group than in the control group in all lobes (all P < 0.001). CT inflammation score and CT fibrosis score were also significantly higher in the experimental group than in the control group (CT inflammation score: 3.60 ± 0.84 vs. 1.20 ± 0.42, P < 0.001; CT fibrosis score: 3.80 ± 0.42 vs. 1.10 ± 1.37, P < 0.001).

**Table 2:** The comparison of CT scores between the experimental and control groups.

	Experimental group	Control group	P-value
**Total CT score**	21.10 ± 6.49	6.60 ± 1.58	< 0.001
**CT inflammation score**	3.60 ± 0.84	1.20 ± 0.42	< 0.001
**CT fibrosis score**	3.80 ± 0.42	1.10 ± 1.37	< 0.001
**CT Scores in each lobe**			
Right, superior lobe	3.10 ± 0.88	1.30 ± 0.48	< 0.001
Right, middle lobe	3.10 ± 0.88	1.10 ± 0.57	< 0.001
Right, inferior lobe	3.10 ± 0.88	1.10 ± 0.32	< 0.001
Postcaval lobe	3.00 ± 0.82	1.00 ± 0.00	< 0.001
Left, upper region	2.90 ± 1.29	0.60 ± 0.52	< 0.001
Left, middle region	3.00 ± 1.05	0.50 ± 0.53	< 0.001
Left, lower region	2.90 ± 0.99	1.10 ± 0.32	< 0.001
**Presence of any CT lesion, n (%)**			
Right, superior lobe	10 (100%)	10 (100%)	N/A
Right, middle lobe	10 (100%)	9 (90%)	> 0.999
Right, inferior lobe	10 (100%)	9 (90%)	> 0.999
Postcaval lobe	10 (100%)	10 (100%)	N/A
Left, upper region	10 (100%)	6 (60%)	0.087
Left, middle region	10 (100%)	5 (50%)	0.033
Left, lower region	10 (100%)	10 (100%)	N/A

### Histopathologic evaluation of inflammation and fibrosis and Western blotting

**Figure 2:**
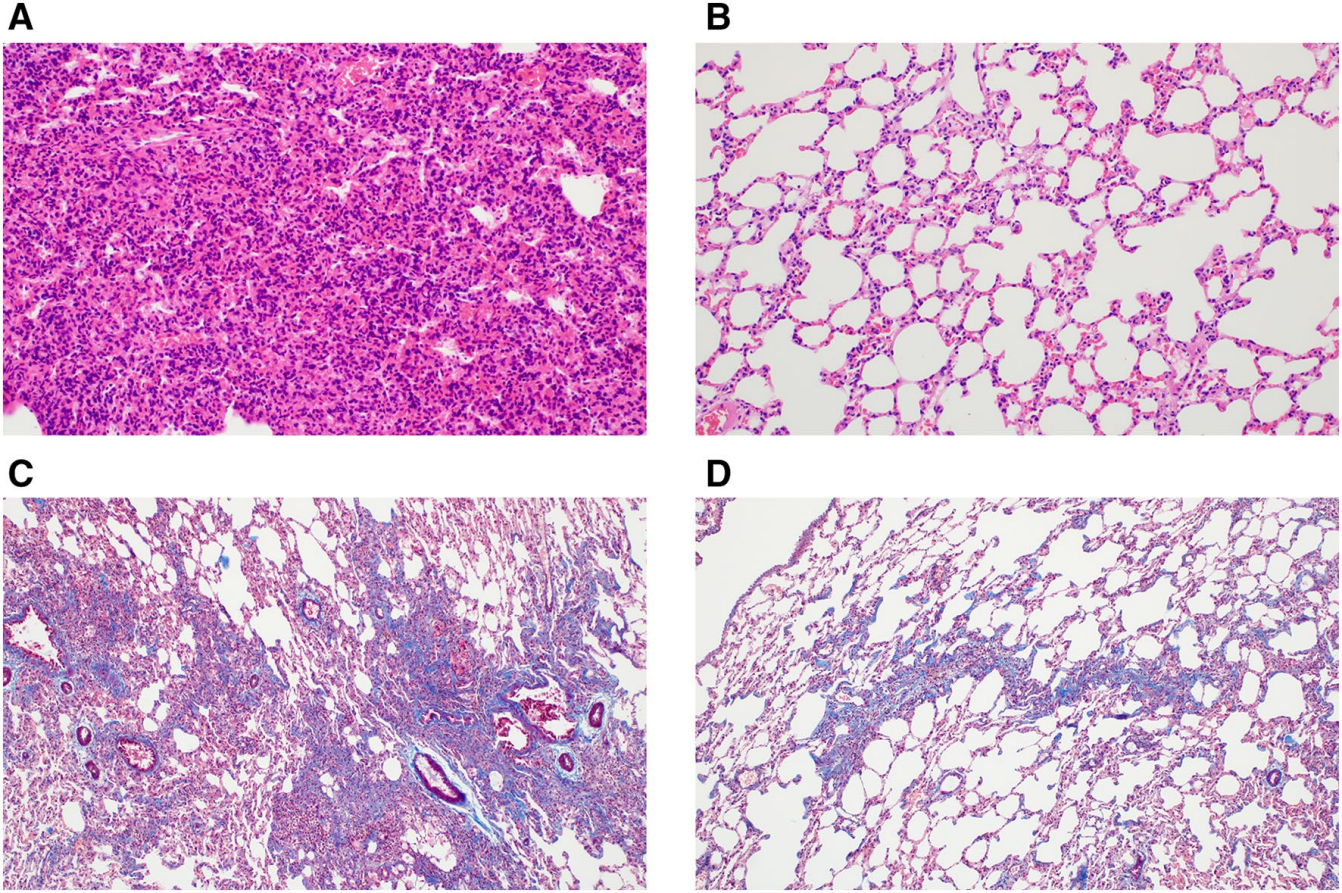
Changes in inflammation and fibrosis between the experimental and control groups. (**A**) Inflammation of the experimental group (X200, hematoxylin and eosin [H&E]), (**B**) inflammation of the control group (X200, H&E), (**C**) fibrosis of the experimental group (X100, Masson’s trichrome [MT]), (**D**) fibrosis of the control group (X100, MT).

Table 3 and Fig. 2 show the results of pathologic analysis for inflammation and fibrosis in both groups. Both the pathologic inflammation score and pathologic fibrosis score were significantly higher in the experimental group than in the control group (pathologic inflammation score: 4.80 ± 1.14 vs. 2.30 ± 0.48, P < 0.001; pathologic fibrosis score: 2.00 ± 1.16 vs. 1.00 ± 1.05, P = 0.042). The extent and severity of inflammation were also significantly higher in the experimental group than the control group (P < 0.001 and P = 0.019, respectively), although there were no significant differences in extent and severity of fibrosis between the two groups.

**Table 3:** The comparison of extent and severity of inflammation and fibrosis in pathology between the experimental and control groups.

	Experimental group	Control group	P-value
**Inflammation extent, n (%)**			
none	0 (0%)	0 (0%)	** < 0.001**
< 25%	0 (0%)	9 (90%)	
25–50%	3 (30%)	1 (10%)	
> 50%	7 (70%)	0 (0%)	
**Inflammation severity, n (%)**			
none	0 (0%)	0 (0%)	**0.019**
mild	5 (50%)	8 (80%)	
moderate	5 (50%)	2 (20%)	
severe	0 (0%)	0 (0%)	
**Pathologic inflammation score**	4.80 ± 1.14	2.30 ± 0.48	** < 0.001**
**Fibrosis extent, n (%)**			
none	2 (20%)	5 (50%)	0.35
< 25%	8 (80%)	5 (50%)	
25–50%	0 (0%)	0 (0%)	
> 50%	0 (0%)	0 (0%)	
**Fibrosis severity, n (%)**			
none	2 (20%)	5 (50%)	0.067
mild	4 (40%)	5 (50%)	
moderate	4 (40%)	0 (0%)	
severe	0 (0%)	0 (0%)	
**Pathologic fibrosis score**	2.00 ± 1.16	1.00 ± 1.05	**0.042**

**Figure 3:**
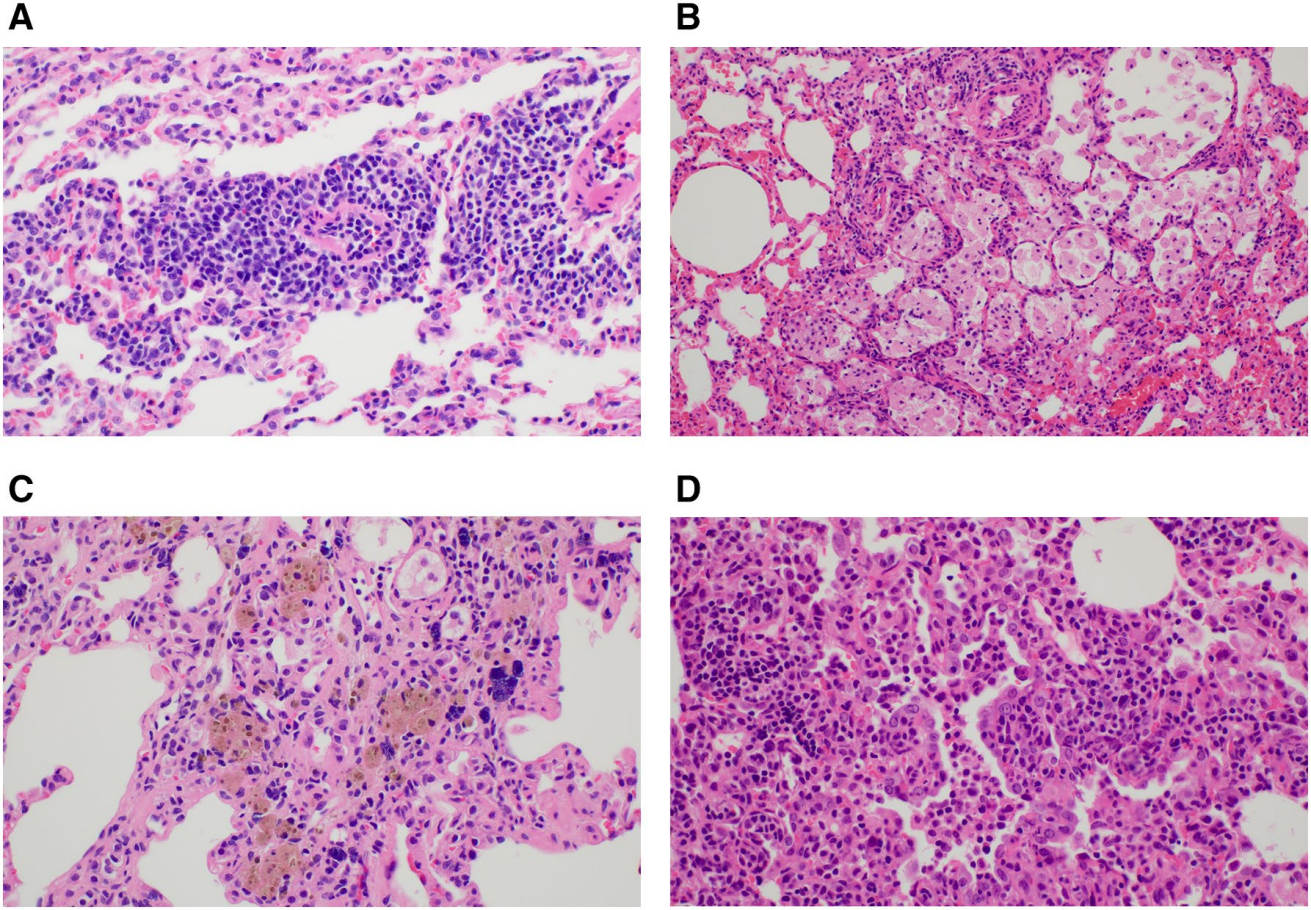
The specific pathologic findings of the experimental group. (**A**) Lymphocytic vasculitis (X400, hematoxylin and eosin [H&E]), (**B**) foamy histiocytes (X200, H&E), (**C**) pigmented macrophages (X400, H&E), (**D**) alveolar atypia (X400, H&E).

The specific pathologic findings are demonstrated in Table 4 and Fig. 3. Lymphocytic vasculitis and foamy histiocytes were more frequently observed in the experimental group than in the control group (P = 0.016 and P = 0.020, respectively). There was no significant difference in the degree of pigmented macrophages and alveolar atypia between the two groups. The expression of myofibroblast protein markers, such as fibronectin and collagen type I, in western blotting was higher in the experimental group than in the control group (Supplementary Figure S1). Among the myofibroblast proteins markers, collagen type I was statistically different between the two groups (P < 0.05).

**Table 4:** The comparison of specific pathologic findings between the experimental and control groups.

	Experimental group	Control group	P-value
**Lymphocystic vasculitis, n (%)**			
none	1 (10%)	7 (70%)	**0.016**
< 25%	1 (10%)	2 (20%)	
25–50%	7 (70%)	1 (10%)	
> 50%	1 (10%)	0 (0%)	
**Foamy histiocyte, n (%)**			
none	1 (10%)	5 (50%)	**0.02**
< 25%	5 (50%)	0 (0%)	
25–50%	4 (40%)	5 (50%)	
> 50%	0 (0%)	0 (0%)	
**Pigmented macrophage, n (%)**			
none	2 (20%)	4 (40%)	0.628
< 25%	8 (80%)	6 (60%)	
25–50%	0 (0%)	0 (0%)	
> 50%	0 (0%)	0 (0%)	
**Alveolar atypia, n (%)**			
none	7 (70%)	10 (10%)	0.171
< 25%	2 (20%)	0 (0%)	
25–50%	1 (10%)	0 (0%)	
> 50%	0 (0%)	0 (0%)

### RNA sequencing analysis and gene ontology (GO) pathway analysis

**Figure 4:**
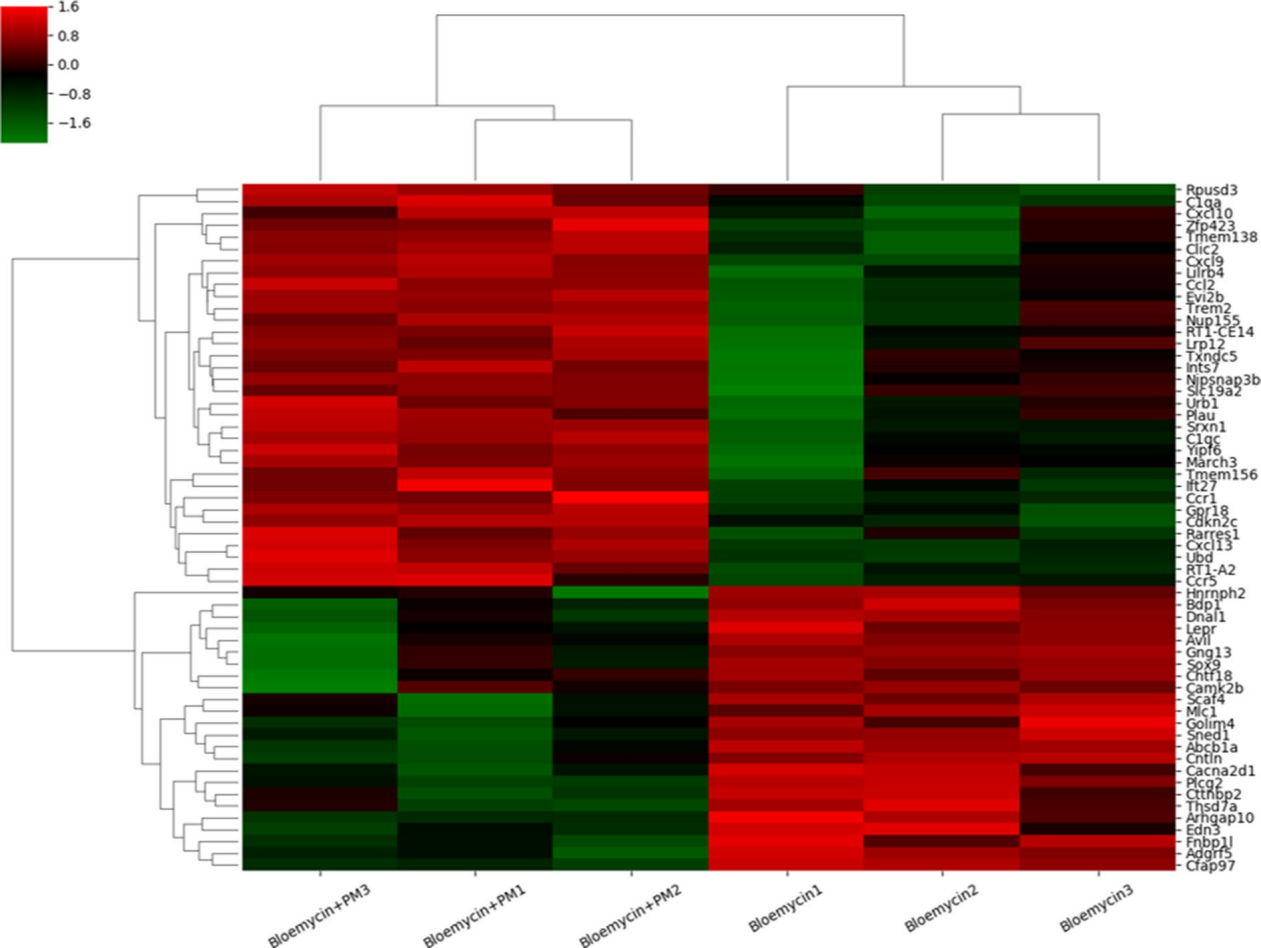
Analysis of particulate matter regulated gene expression in rat lung tissue. Heatmap of particulate matter in 56 regulated genes (> 2 or < -2 folds, P < 0.05) based on gene clustering of QuantSeq 3’ mRNA-Seq results.

A total of 56 genes out of 17,048 expressed significant changes in PM-treated lung tissue with a standard P-value < 0.05 and log > 2 or < -2. Among these, 32 genes were upregulated and 24 genes were downregulated in PM-treated lung tissue compared to control lung tissue (Fig. 4). Supplementary Tables 1 and 2 summarize these 56 significantly altered genes. The CCL2, CXCL9, CXCL10, CXCL13, CCR5, and CCR1 genes, associated with inflammation and immunity, were upregulated. In addition, the mRNA levels of CCL2, CXCL9, CXCL10, and CXCL13 were upregulated in the PM-treated lung tissue using real-time PCR (Supplementary Figure S2).

**Figure 5:**
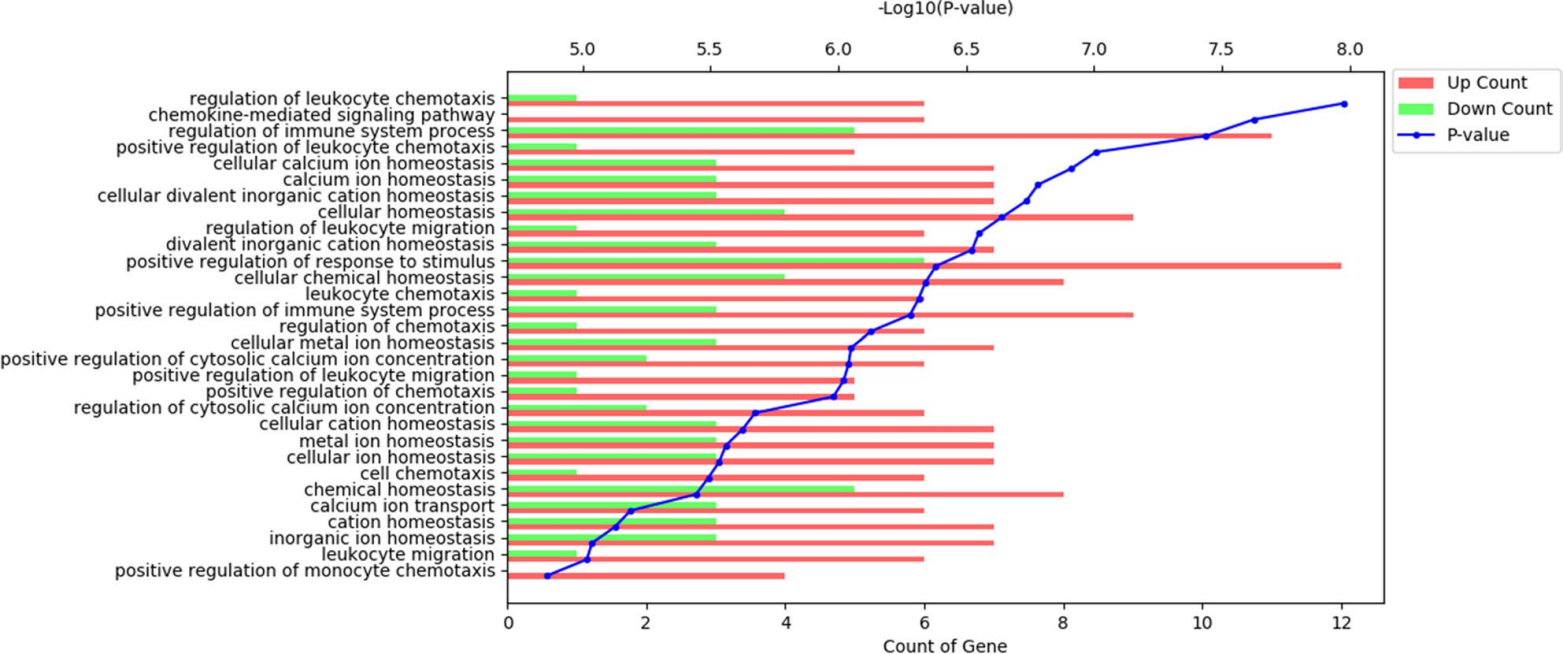
Gene ontology (GO) pathway analysis of differentially expressed genes in the control group versus the experimental group.

The differentially expressed genes (DEGs) were significantly enriched in the 30 GO biologic processes (P < 0.05) (Fig. 5). Many genes that respond to the leukocyte chemotaxis/migration and calcium ion/inorganic cation/chemical/metal ion homeostasis were prominently reported in most of the GO pathway analyses.

## Discussion

The present study showed that chronic PM exposure aggravated pulmonary fibrosis and inflammation underlying bleomycin-induced lung fibrosis, as proven by chest CT, pathological analysis, and RNA sequencing. Inflammation and fibrosis scores in both chest CT and pathologic analysis were significantly more aggravated in a bleomycin-induced lung fibrosis rat model with chronic PM exposure than without PM exposure. In addition, several genes associated with inflammation and immunity were upregulated in a bleomycin-induced lung fibrosis rat model with chronic PM exposure.

Bleomycin-induced lung fibrosis resembles human fibrotic lung disease, such as IPF, due to some molecular signatures or histopathologic hallmarks^17–19^. IPF is a specific form of chronic, progressive, fibrosing interstitial pneumonia of unknown cause^20^. IPF is characterized by progressive worsening of dyspnea and lung function and is associated with a poor prognosis^20^. Some recent epidemiological studies have used a statistical model to examine the effect of ambient PM on IPF patients^21–25^. Johannson et al. reported an association between the risk of acute IPF exacerbation and air pollution exposure, including ozone (O_3_), nitrogen dioxide (NO_2_), and PM_2.5_/PM_10_^21^. Conti et al. also observed an association between chronic exposure to air pollution and increased incidence of IPF^22^. In addition, PM exposure was significantly associated with increased mortality and decreased forced vital capacity in patients with IPF^23,24^.

There have been several animal reports that indicate PM increases the severity of bleomycin-induced pulmonary fibrosis and inflammation. Kamata et al. showed that carbon black nanoparticles aggravated bleomycin-induced lung inflammation and fibrosis in mice three weeks after a one-time exposure to bleomycin plus carbon black^26^. Cheng et al. found that one instillation of bleomycin plus PM collected in the Washington, D.C. area aggravated inflammation and fibrosis two weeks after exposure^4^. Xu et al. demonstrated that PM_2.5_ collected in China enhanced bleomycin-induced fibrosis two weeks after instillation by stimulating endoplasmic reticulum stress in rats^5^. All of these experiments involved simultaneously administrated PM and bleomycin and only demonstrated the short-term exposure effect of PM.

However, the experimental design of our study differed from previous studies. To analyze the effect of chronic PM exposure on bleomycin-induced fibrosis, we exposed rats to PM once a week for 10 weeks, whereas only one instillation of PM was performed in previous studies. In addition, PM instillation was performed four weeks after bleomycin administration in our study to evaluate the formation of evident fibrosis. It is known that intra-alveolar and septal fibrosis become morphologically evident three or four weeks post-bleomycin, which is the chronic fibrosis stage^18^. The acute injury and inflammatory phase begins, within one week after the bleomycin injection^27^. One to two weeks after bleomycin, there is a gradual subsidence of the inflammatory response with an accompanying increase in fibrosis^28^. As it would be difficult to assess inflammation or fibrosis caused by PM at the acute stage, PM was additionally administered at the chronic fibrosis stage in our experiment. However, some studies reported that the fibrotic lesions resolved after days 21–28^27,29^. In our study, 50% of the control group did not show bronchiectasis and linear densities suggesting lung fibrosis in CT analysis, whereas 100% of the experimental group showed bronchiectasis and linear densities. In pathologic analysis, the pathologic fibrosis score of the experimental group was significantly higher than the pathologic fibrosis score of the control group. Based on these results, it is presumed that chronic exposure to PM interferes with the recovery of bleomycin-induced lung fibrosis and causes persistent inflammation and fibrosis exacerbation. Unlike bleomycin-induced lung fibrosis in the murine model which may show recovery, lung fibrosis, such as IPF in humans, shows continuous exacerbation. Therefore, chronic exposure to PM may be more lethal in patients with lung fibrosis.

In our study, chest CT also reflected significantly increased lung fibrosis and inflammation in a bleomycin rat model after PM exposure, which was correlated with pathologic findings and western blotting. Several studies have evaluated chest CTs in a rat model, and CT findings in rat lung tissue evaluated by a radiologist have correlated with pathologic features^12^. A previous study showed that CT findings, such as linear densities indicating focal or multifocal segmental atelectasis showing linear configuration and bronchiectasis indicating bronchial dilatation with a lack of tapering of the bronchi, were pathologically fibrosis or inflammation^12^. Based on these results, CT outcomes in a rat lung model with bleomycin plus PM could be used to analyze human chest CT findings.

In our RNA sequencing analysis, several chemokine ligand/receptor genes associated with inflammation and the immune system were significantly increased in the PM exposure group. Chemokine C-X-C motif ligand 13 (CXCL13) and its receptor (CXCR5) play fundamental roles in inflammatory, infectious, and immune reactions. Bracke et al. reported that CXCL 13 was increased in the lungs of chronic cigarette smoke-exposed mice and COPD patients^30^. Chemokine C–C motif ligand 2 (CCL2) reveals a chemotactic activity for monocytes and basophils. A recent study showed that CCL2 was upregulated in the lung tissue of PM-exposed rats in a dose-dependent manner^31^. Chemokine C-X-C motif ligand 9 (CXCL9) and its receptor (CXCR3) regulate immune cell migration, differentiation, and activation. Interestingly, CXCL9 is elevated in the lungs of acute cigarette smoke-exposed mice and patients with COPD^32,33^.

There were several limitations in this study. First, RNA sequencing was performed for only three rats, not in all of the rats in each group. However, because the primary goal of this study was to show the effect of chronic PM exposure in bleomycin-induced lung fibrosis using chest CT and pathologic analysis, most of the tissue could not be utilized for RNA sequencing. Second, saline buffer with extracted PM was filtered using a 0.2 μm syringe filter to remove the quartz mixed into the PM suspension. A 0.2 μm syringe filter was used to exclude the influence of the quartz component on the lungs, but some other PM components may have been removed during this process. However, we listed and analyzed the PM components used in this experiment in the supplementary tables.

In conclusion, chronic PM exposure aggravated pulmonary fibrosis and inflammation underlying bleomycin-induced lung fibrosis. These findings were proven by chest CT and pathologic analysis. Genetic alterations due to PM exposure may provoke pulmonary inflammation.

## Methods

This study was approved by the Institutional Animal Care and Use Committee of the Korea University Medical Center (Approval number: Korea-2020–0025-C1). This study was carried out in compliance with the ARRIVE guidelines, and all experiments were performed per Korea University guidelines.

### Animals

Nine-week-old male Sprague–Dawley rats (Raonbio, Yong-in, South Korea) were acclimated for one week (three rats per cage). The conditions were as follows: temperature, 22–25℃; relative humidity, 40–60%; and lighting conditions, 12 h light/dark cycles. Pelleted food for experimental rodents (Purina, Sung-nam, South Korea) and filtered tap water were provided ad libitum.

### Collecting filtered liquid fine PM

A high-volume air sampler (HV-1700RW, SIBATA, Tokyo, Japan) with a quartz filter (QR-100, Sibata) was used to collect atmospheric PM five days a week at a flow rate of 1000 L/min on the rooftop of Korea University Ansan Hospital, in Gyeonggi-province, South Korea. Before extracting PM, the filters were dried in an auto-desiccator (Sanpla Dry Keeper, Sanplatec Co., Osaka, Japan). As one filter was used per day for air sampling, we selected seven filters above the WHO guidelines for PM_2.5_ (average of 25 μg/m^3^ or more per day) and PM_10_ (average of 50 μg/m^3^ or more per day). We referred to Real-Time Air Quality Data (https://www.airkorea.or.kr/eng), dated November 5, 2018, November 6, 2018, November 27, 2018, January 15, 2019, January 22, 2019, March 26, 2019, and March 27, 2019.

To prepare the PM suspension, we collected quartz filters cut into 2 cm × 2 cm pieces and immersed them in 210 ml of a 0.9% saline buffer, and then sonicated them for 30 min. After shaking for 10 min, we filtered the saline buffer with a 0.2 μm syringe filter to remove the quartz component in the PM suspension. Supplementary Table 3 describes the final components of the PM suspension.

### Experimental design

A total of 20 rats were randomly divided into two groups: the bleomycin + PM treated group (experimental group, 10 rats) and the bleomycin + sterile phosphate-buffered saline (PBS) treated group (control group, 10 rats). All rats were anesthetized with 2% isofluorane in 70% N_2_O and 30% O_2_ for intratracheal instillation of bleomycin, PBS, and PM. A solution of bleomycin (1.5 mg/kg) was intratracheally administrated to the rats under the guidance of a modified videoscope for intratracheal instillation. After four weeks of bleomycin treatment, 150 uL of PM suspension or normal saline was intratracheally administrated to the rats under the guidance of a modified videoscope every week for 10 weeks (one instillation [150 uL] per week × 10 = 1.5 mL). The respiration volume of a person (60 kg) is generally 9,660 L/day (https://www.epa.gov/expobox/exposure-assessment-tools-routes), and the suction volume of an air sampler is 1,380,000 L/day. Therefore, the amount of airflow extracted by the air sampler over seven days is equal to the respiration volume of a human over 1,000 days (1,380,000 L/day × 7 days / 9,660 L/day = 1,000 days). In this study, seven filters collected for seven days were extracted into 210 mL of a saline buffer. As this is the same amount humans are exposed to over 1,000 days, it was assumed that 0.210 mL of PM suspension is the daily human exposure (210 mL/1,000 days = 0.210 mL/day), and human exposure in one day (24 h) was assumed to be 0.21 mL. The total amount administrated to rats (1.5 mL) was assumed to be the same as the human PM exposure in 7.14 days or 171.4 h (1.5 mL / 0.21 mL/day = 7.14 days; 7.14 days × 24 h/day = 171.4 h).

**Figure 6:**
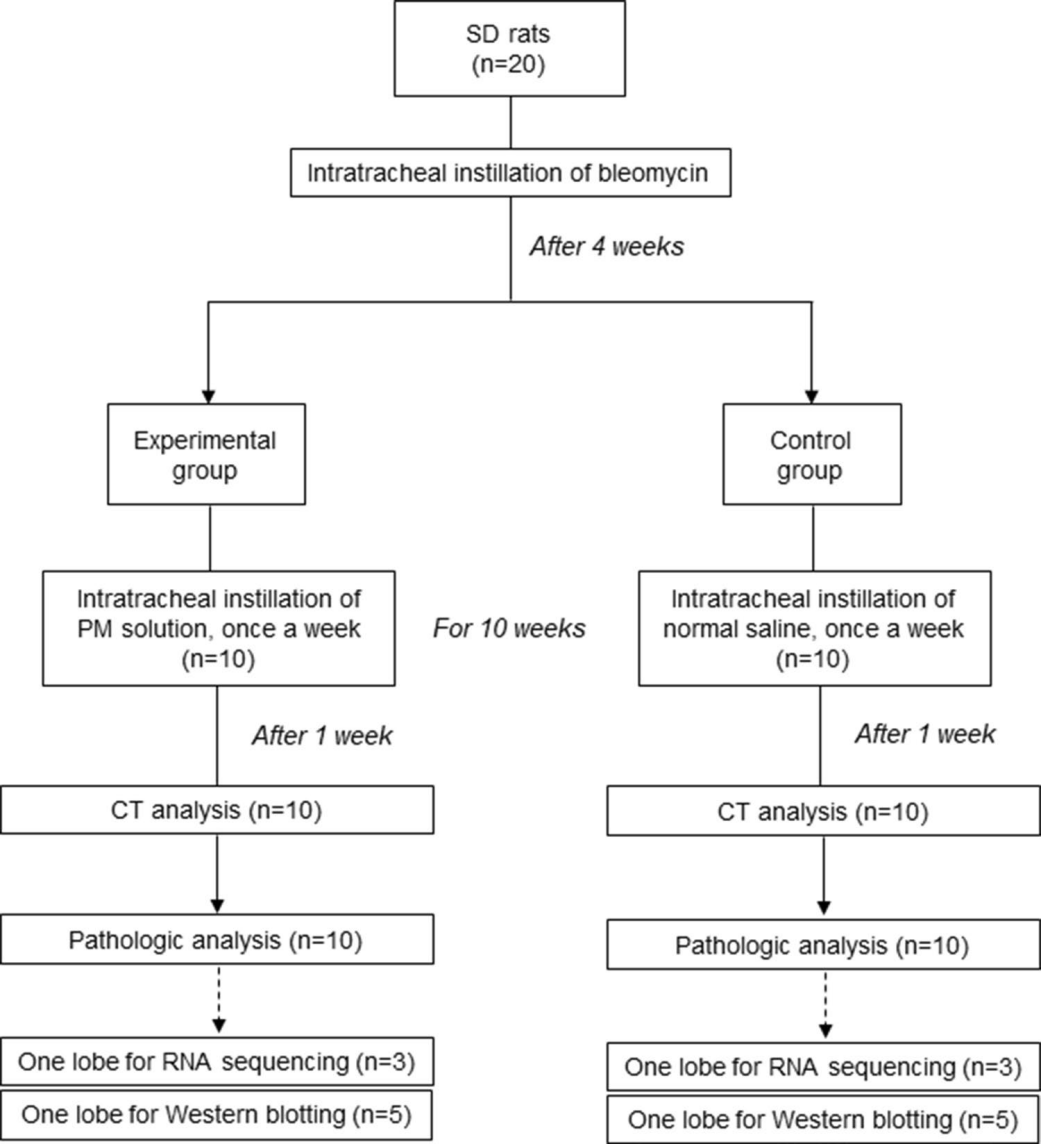
Summary of the experimental design.

One week after the final intratracheal instillation, all rats underwent a CT examination under anesthetic conditions with an intraperitoneal and intramuscular injection of Alfaxan (30 mg/kg) and Xylazine (10 mg/kg), respectively. Subsequently, the animals were sacrificed and both lungs were collected for histopathological evaluation. The experimental design is summarized in Fig. 6. There were no deaths during the experiment.

### CT protocol

All CT images were scanned using a Philips IQon 128-slice dual-layer detector spectral CT scanner (Philips Healthcare, Cleveland, OH, USA) with the subject in the supine position. All images were obtained in a caudo-cranial direction from the lung base through the thoracic inlet level during inspiration breath-hold using a ventilator for small animals (VentElite, Harvard Apparatus, MA, USA). The scan time for one rat was less than 10 s. CT scan parameters were as follows: kVp, 80; mA, 400; collimation, 64 × 0.625 mm; slice thickness, 0.67 mm; beam width, 40 mm; pitch, 1.048; and rotation time, 0.4 s.

### Quality analysis of CT images

One board-certified radiologist with 11 years of experience in thoracic imaging (C.K.) analyzed the CT image quality to determine if it was suitable for evaluating the rat lung tissue. The image quality was evaluated on a five-point scale: 1: severely impaired detectability of CT findings; 2: moderately impaired detectability of CT findings; 3: mildly impaired detectability of CT findings; 4: mildly impaired detectability of CT findings, but no significant impairment; and 5: unimpaired detectability of CT findings with high confidence in the diagnosis.

### CT evaluation

One board-certified radiologist with 11 years of experience in thoracic imaging (C.K.) who was blinded to the groups reviewed all of the CT images. CT findings, including consolidation, ground-glass opacity (GGO), centrilobular nodules, and bronchiectasis/linear densities, were evaluated and followed or modified according to the glossary of radiologic terms for human chest CT suggested by the Fleischner Society (Supplementary Table 4)^11^ and previous studies^12^. Among the CT findings, consolidation, GGO, and centrilobular nodules were considered “inflammation”, and bronchiectasis/linear densities were considered “fibrosis”. These CT findings were evaluated in the four right lung lobes (superior, middle, inferior, and post-caval lobes) and the three regions of the left lung (upper region, middle region, and lower region). The extent of the four CT findings (consolidation, GGO, centrilobular nodules, and bronchiectasis/linear densities) in each lobe/region was scored (0 = none, 1 = lesions involving 1–25% of a lobe, 2 = lesions involving 26–50% of a lobe, 3 = lesions involving 51–75% of a lobe, 4 = lesions involving 76–100% of a lobe). The total CT score was defined as the sum of the score of any CT findings in the bilateral lungs, and the CT parameter scores were defined as the sum of the scored extent of CT findings for inflammation or fibrosis in the bilateral lungs. The CT inflammation score was defined as the sum of the CT score for consolidation, GGO, nodules, and centrilobular nodules. The CT fibrosis score was the sum of the CT score of the bronchiectasis/linear densities.

### Histologic examination

All extracted lung specimens were evaluated by one experienced pathologist with 21 years of experience in thoracic pathology (J.L.). The lungs were fixed in 10% neutral buffered formalin. We cut 4-um-thick paraffin sections from the fixed samples and then performed hematoxylin and eosin (H&E) and Masson’s trichrome (MT) staining. After collecting a small amount of frozen lung tissue for RNA-sequence testing and western blotting, slides were made for all lung tissue in 1-mm intervals.

We evaluated the extent (none, lesions involving < 0–25%/ < 25–50%/ > 50% of the total lung area) and severity (none/mild/moderate/severe) of inflammation and fibrosis. The inflammation and fibrosis pathologic scores were calculated by adding the extent and severity of inflammation and fibrosis, with extent scored as 0 = none, 1 = lesions involving < 0–25% of the lung, 2 = lesions involving < 25–50% of the lung, and 3 = lesions involving > 50% of the lung) and severity as 0 = none, 1 = mild, 2 = moderate, and 3 = severe. The presence of pathologic findings, such as lymphocytic vasculitis, foamy histiocyte, pigmented macrophages, and alveolar cell atypia were evaluated in each group.

### RNA isolation and real-time PCR

One lobe in each rat (three rats in each group) was selected after the radiologist reviewed the correlated chest CTs. The radiologist chose lobes with lesions similar to those of the other lobes. Lobes with no or too few lesions were excluded. Then, the selected lobes were ground up and lysed using a lysis buffer containing 2-mercaptoethanol. The total RNA was isolated using Trizol reagent (Invitrogen, Carlsbad, CA). The RNA quality was assessed by an Agilent 2100 Bioanalyzer (Agilent Technologies, Santa Clara, CA, USA) using the RNA 6000 Nano Chip (Agilent Technologies, Amstelveen, The Netherlands), and RNA quantification was performed using a NanoDrop 2000 spectrophotometer (Thermo Scientific, Wilmington, DE, USA). For gene expression experiments, real-time PCR was performed in a LightCycler 96 system (Roche Life Science, Branford, CT, USA) with the following primers: CCL2; 5'-AGCCAACTCTCACTGAAGCC-3' (sense), 5'-AACTGTGAACAACAGGCCCA-3' (antisense), CXCL9; 5'-GTTTGCCCCAAGCCCTAACT-3' (sense), 5'-GCTGAATCTGGGTCTAGGCA-3' (antisense), CXCL10; 5'-GGCCTGGTCCTGAGACAAAA-3' (sense), 5'-CTGCCTGAGGGAAGATTCGG-3' (antisense), CXCL13; 5'-TGTAGGTGTTCCAAGGTGAGC-3' (sense), and 5'-CAGTTTTGGGGCAGCCATTC-3' (antisense). All samples were performed in duplicate, and the gene levels were statistically analyzed by a t-test.

### Western blotting

One lobe in each rat (five rats in each group) was selected after the radiologist reviewed the correlated chest CT. The radiologist chose lobes with lesions similar to those in the other lobes. Lung tissues were lysed in a T-PER™ Tissue Protein Extraction Reagent (Thermo Scientific, Rockford, IL, USA) using a homogenizer (OMNI International, Waterbury, CT, USA). Equal amounts of protein extracts (20 μg) were separated by sodium dodecyl sulfate–polyacrylamide gel electrophoresis using a natural gel and then transferred onto a polyvinylidene fluoride membrane (Atto, Tokyo, Japan). After blocking with 5% non-fat skim milk for one hour, the membrane was incubated overnight with rabbit anti-collagen type I (1:1,000, Abcam, Cambridge, UK), rabbit anti-fibronectin (1:1,000, Abcam), and mouse β-actin (1:5,000, Santa Cruz, CA, USA). Afterward, an appropriate horseradish peroxidase-conjugated anti-rabbit IgG or anti-mouse IgG antibody (Cell Signaling Technology, Danvers, MA, USA) was used to bind to the primary antibodies. Protein band imaging was done using the ChemiDoc Touch Imaging System (Bio-Rad Laboratories).

### Library preparation and sequencing

The library for the control and test RNAs was constructed using a QuantSeq 3’ mRNA-Seq Library Prep Kit (Lexogen, Inc., Austria) per the manufacturer’s instructions. In brief, 500 ng of the total RNA was prepared and an oligo-dT primer containing an Illumina-compatible sequence at its 5’ end was hybridized to the RNA. Reverse transcription was then performed. After we degraded the RNA template, we initiated the second strand synthesis by a random primer containing an Illumina-compatible linker sequence at its 5’ end. The double-stranded library was purified with magnetic beads to remove all reaction components. The library was amplified to add the complete adapter sequences required for cluster generation. The finished library was purified from the PCR components, and high-throughput sequencing was performed as single-end 75 sequencing using a NextSeq 500 (Illumina, Inc., USA).

### Data analysis

QuantSeq 3′ mRNA-Seq reads were aligned using Bowtie2^34^. Bowtie2 indices were generated either from the genome assembly sequence or the representative transcript sequences for alignment to the genome and transcriptome. The alignment file was used to assemble transcripts, estimate their abundances, and detect differential gene expression. Differentially expressed genes were determined based on counts from unique and multiple alignments using coverage in Bedtools^35^. The read count (RC) data was processed based on the quantile normalization method using EdgeR and within R using Bioconductor^36,37^. The gene lists were further analyzed in the Gene Ontology database to identify expressed genes with similar functions through the DAVID (http://david.niaid.nih.gov) and Medline databases (http://www.ncbi.nlm.nih.gov/). We identified the genes whose expression was significantly changed (standard P-value < 0.05 and log > 2 or < -2) by PM exposure.

### Gene ontology (GO) pathway analysis

We used a web-based genome enrichment analysis tool to comprehensively elucidate the functional classification of the DEGs. P < 0.05 was used as a threshold for the significantly enriched GO terms.

### Statistical analysis

The chi-square test for nominal variables and the Mann–Whitney U test for continuous variables were performed to determine differences between CT features and pathologic findings among the groups. All statistical analyses were performed using SPSS Statistics 20 (SPSS, Chicago, IL, USA) or MedCalc version 18.5 (MedCalc Software, Ostend, Belgium). All P-values < 0.05 were considered statistically significant.

## Supplementary Information

Supplementary Information.

## Data Availability

All data generated or analyzed during this study are included in this published article.

## Abbreviations

PM: Particulate matter
IPF: Idiopathic pulmonary fibrosis
GGO: Ground-glass opacity
