# Supplementary material for: Evaluation of the effect of filtered ultrafine particulate matter on bleomycin-induced lung fibrosis in a rat model using computed tomography, histopathologic analysis, and RNA sequencing
Source: Sci Rep. 2021 Nov 22;11:22672. doi: 10.1038/s41598-021-02140-2 (PMC8609022; doi:10.1038/s41598-021-02140-2)
Supplement: Supplementary file 1 — Supplementary Information. [file 41598_2021_2140_MOESM1_ESM.docx]

**Evaluation of the effect of filtered ultrafine particulate matter on bleomycin-induced lung fibrosis in a rat model using computed tomography, histopathologic analysis, and RNA sequencing**

Cherry Kim^1,^*, Sang Hoon Jeong^2,^*, Jaeyoung Kim^2^, Ja Young Kang^2^, Yoon Jeong Nam^2^, Ariunaa Togloom^2^, Jaehyung Cha^2^, Ki Yeol Lee^1^, Chang Hyun Lee^3^, Eun-Kee Park^4^, Ju-Han Lee^5^

* These authors have equally contributed to this study.

^1^Department of Radiology, Ansan Hospital, Korea University College of Medicine, 123, Jeokgeum-ro, Danwon-gu, Ansan-si, Gyeonggi, 15355, South Korea

^2^Medical Science Research Center, Ansan Hospital, Korea University College of Medicine, 123, Jeokgeum-ro, Danwon-gu, Ansan-si, Gyeonggi, 15355, South Korea

^3^Department of Radiology, College of Medicine, Seoul National University, Seoul National University Hospital, Seoul, 03080, South Korea

^4^Department of Medical Humanities and Social Medicine, College of Medicine, Kosin University, Busan, 49267, South Korea

^5^Department of Pathology, Ansan Hospital, Korea University College of Medicine, 123, Jeokgeum-ro, Danwon-gu, Ansan-si, Gyeonggi, 15355, South Korea

**Corresponding author:** Ju-Han Lee, MD, Ph.D.

Department of Pathology, Ansan Hospital, Korea University College of Medicine, 123, Jeokgeum-ro, Danwon-gu, Ansan-si, Gyeonggi, 15355, South Korea

E-mail: [repath@korea.ac.kr](mailto:repath@korea.ac.kr), Phone: 82-31-412-5322, Fax: 82-31-412-5324

**Supplementary Figure S1.** (A) Western blotting results, and (B) the raw data of western blotting results.

(A)


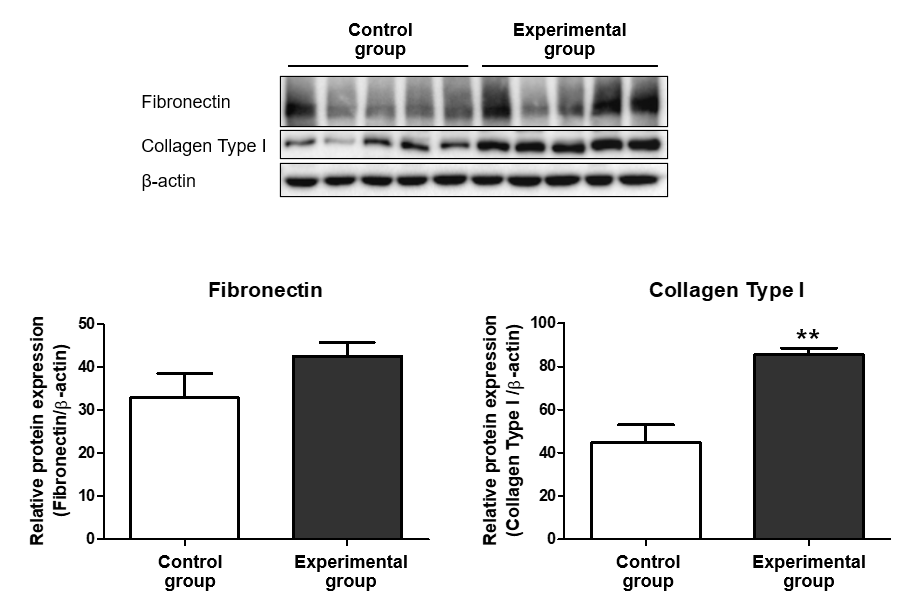


(B)


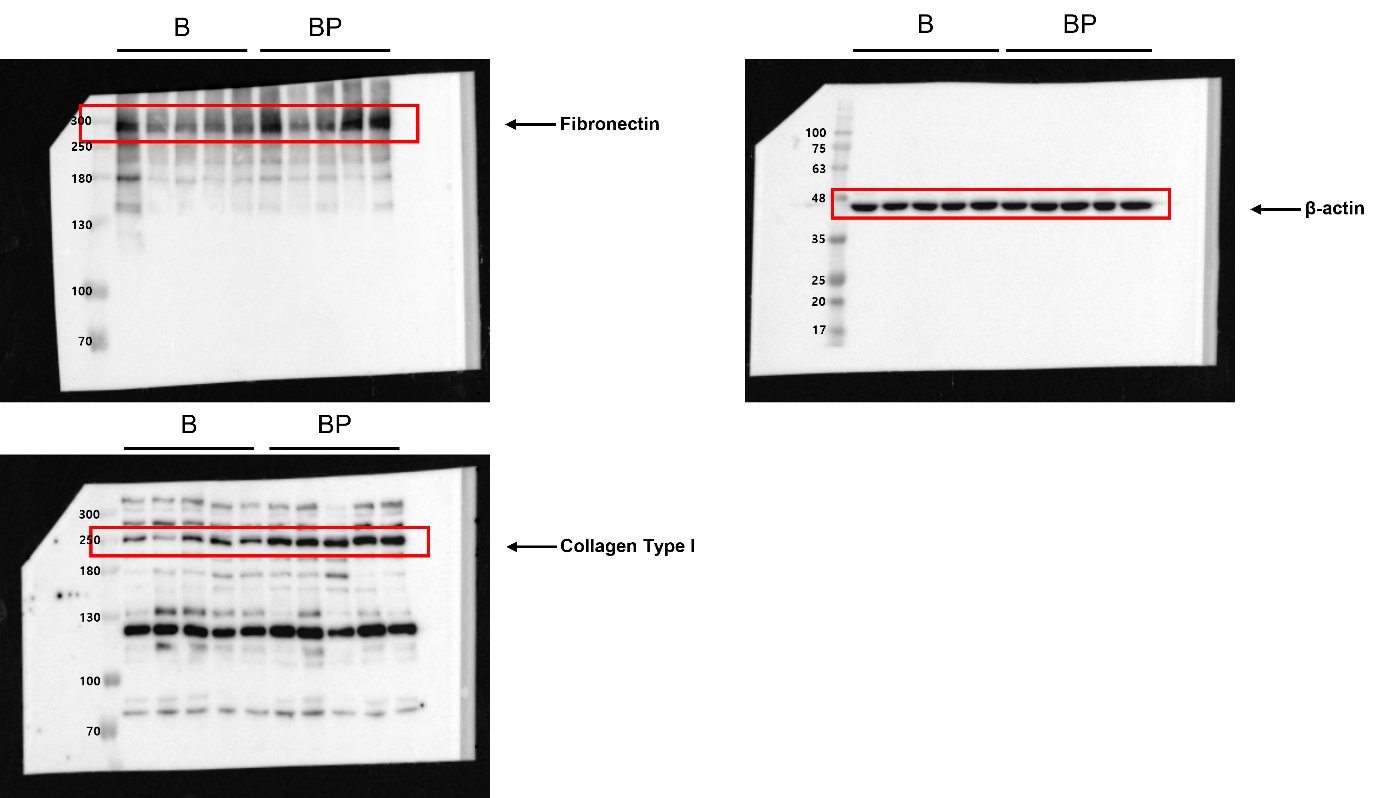


**Supplementary Figure S2.** The result of the real-time PCR analysis of CCL2, CXCL9, CXCL10, and CXCL13. The expression levels were normalized with glyceraldehyde 3-phosphate dehydrogenase (GAPDH).


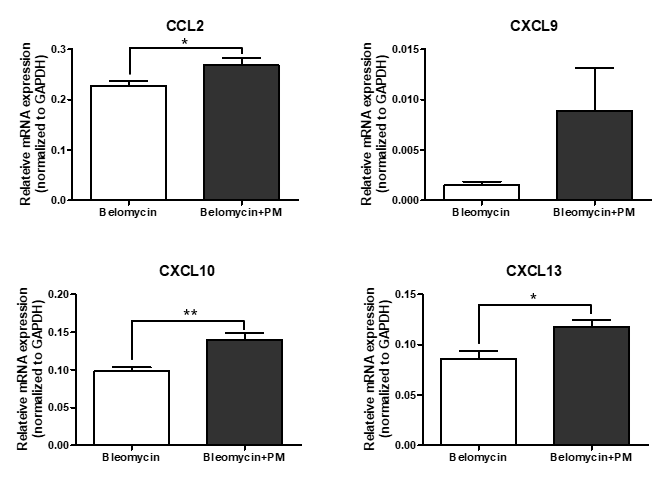


*P<0.05

**P<0.01

**Supplementary Table 1.** The list of up-regulated genes in the particulate matter treated group (> 2 folds, p < 0.05)

| **Gene symbol** | **Description** | **Fold change** | **P-value** |
| --- | --- | --- | --- |
| Cxcl13 | C-X-C motif chemokine ligand 13 | 8.872 | 0.010 |
| Ubp | ubiquitin D | 8.614 | 0.017 |
| Ccl2 | C-C motif chemokine ligand 2 | 6.702 | 0.012 |
| Cxcl10 | C-X-C motif chemokine ligand 10 | 3.806 | 0.048 |
| Cxcl9 | C-X-C motif chemokine ligand 9 | 3.689 | 0.005 |
| Ccr5 | chemokine (C-C motif) receptor 5 | 2.967 | 0.042 |
| Lilrb4 | leukocyte immunoglobulin like receptor B4 | 2.789 | 0.006 |
| Urb1 | URB1 ribosome biogenesis 1 homolog | 2.723 | 0.032 |
| Lrp12 | LDL receptor related protein 12 | 2.589 | 0.043 |
| Rpusd3 | RNA pseudouridylate synthase domain containing 3 | 2.572 | 0.021 |
| Ccr1 | C-C motif chemokine receptor 1 | 2.551 | 0.027 |
| Tmem138 | transmembrane protein 138 | 2.541 | 0.009 |
| Nipsnap3b | nipsnap homolog 3B | 2.518 | 0.016 |
| C1qa | complement C1q A chain | 2.395 | 0.010 |
| Srxn1 | sulfiredoxin 1 | 2.394 | 0.001 |
| Evi2b | ecotropic viral integration site 2B | 2.394 | 0.003 |
| Tmem2 | triggering receptor expressed on myeloid cells 2 | 2.367 | 0.019 |
| Gpr18 | G protein-coupled receptor 18 | 2.354 | 0.001 |
| Nup155 | nucleoporin 155 | 2.351 | 0.024 |
| Yipf6 | Yip1 domain family, member 6 | 2.331 | 0.014 |
| Slc19a2 | solute carrier family 19 member 2 | 2.329 | 0.048 |
| Txndc5 | thioredoxin domain containing 5 | 2.266 | 0.026 |
| Zfp423 | zinc finger protein 423 | 2.264 | 0.035 |
| Plau | plasminogen activator, urokinase | 2.227 | 0.042 |
| Tmem156 | transmembrane protein 156 | 2.208 | 0.034 |
| Ift27 | intraflagellar transport 27 | 2.157 | 0.024 |
| C1qc | complement C1q C chain | 2.135 | 0.002 |
| Clic2 | chloride intracellular channel 2 | 2.125 | 0.004 |
| March3 | membrane associated ring-CH-type finger 3 | 2.112 | 0.013 |
| Rarres1 | retinoic acid receptor responder 1 | 2.085 | 0.023 |
| Cdkn2c | cyclin-dependent kinase inhibitor 2C | 2.023 | 0.001 |
| Ints7 | integrator complex subunit 7 | 2.018 | 0.049 |

**Supplementary Table 2.** The list of down-regulated genes in the particulate matter treated group (<-2 folds, P < 0.05)

| **Gene symbol** | **Description** | **Fold change** | **P-value** |
| --- | --- | --- | --- |
| Golim4 | golgi integral membrane protein 4 | 0.499 | 0.030 |
| Adgrf5 | adhesion G protein-coupled receptor F5 | 0.497 | 0.005 |
| Scaf4 | SR-related CTD-associated factor 4 | 0.487 | 0.015 |
| Plcg2 | phospholipase C, gamma 2 | 0.482 | 0.002 |
| Cacna2d1 | calcium voltage-gated channel auxiliary subunit alpha2delta 1 | 0.470 | 0.021 |
| Fnbp1l | formin binding protein 1-like | 0.468 | 0.016 |
| Lepr | leptin receptor | 0.459 | 0.022 |
| Cfap97 | cilia and flagella associated protein 97 | 0.441 | 0.001 |
| Arhgap10 | Rho GTPase activating protein 10 | 0.440 | 0.019 |
| Cttnbp2 | cortactin binding protein 2 | 0.436 | 0.033 |
| Edn3 | endothelin 3 | 0.433 | 0.046 |
| Chtf18 | chromosome transmission fidelity factor 18 | 0.432 | 0.035 |
| Bdp1 | B double prime 1, subunit of RNA polymerase III transcription initiation factor IIIB | 0.415 | 0.011 |
| Hnrnph2 | heterogeneous nuclear ribonucleoprotein H2 (H') | 0.408 | 0.029 |
| Sned1 | sushi, nidogen and EGF-like domains 1 | 0.406 | 0.003 |
| Camk2b | calcium/calmodulin-dependent protein kinase II beta | 0.363 | 0.045 |
| Gng13 | G protein subunit gamma 13 | 0.355 | 0.008 |
| Abcb1a | ATP binding cassette subfamily B member 1A | 0.338 | 0.001 |
| Dnal1 | dynein, axonemal, light chain 1 | 0.334 | 0.004 |
| Thsd7a | thrombospondin type 1 domain containing 7A | 0.325 | 0.042 |
| Sox9 | SRY box 9 | 0.315 | 0.008 |
| Cntln | centlein | 0.310 | 0.003 |
| Mlc1 | megalencephalic leukoencephalopathy with subcortical cysts 1 | 0.256 | 0.027 |
| Avil | advillin | 0.237 | 0.006 |

**Supplementary Table 3.** The components of particulate matter and the control buffer.

| **Component (unit)** | **Particulate matter extracted from filter** | **Normal saline with blank filter** |
| --- | --- | --- |
| NO_3_^-^ (ppm) | 1865.000 | 6.528 |
| SO_4_^3-^ (ppm) | 568.100 | 8.763 |
| NH_4_^+^ (ppm) | 487.700 | Not detected |
| K^+^ (ppm) | 46.570 | Not detected |
| Ca^2+^ (ppm) | 160.000 | 12.070 |
| Water soluble Carbon (ppm) | 226.000 | 21.000 |
| Mn (ug/L) | 2653 | 33 |
| Fe (ug/L) | 1801 | 35 |
| Ni (ug/L) | 200 | 31 |
| Cu (ug/L) | 1290 | 37 |
| As (ug/L) | 290 | <1 |
| Cd (ug/L) | 82 | <1 |
| Pb (ug/L) | 172 | 3 |

**Supplementary Table 4**. The definition of CT findings from the glossary of radiologic terms for human chest CT suggested by the Fleischner Society.

| **CT findings** | **Definition** |
| --- | --- |
| Consolidation | Homogeneous increase in parenchymal attenuation obscuring the margins of the vessel and airway walls |
| Ground-glass opacity (GGO) | Hazy increased lung opacities with the preservation of bronchial/vascular margins |
| Centrilobular nodule | Nodules which appeared to be separated from the pleural surfaces, fissures, and interlobular septa |
| Bronchiectasis | Bronchial dilatation with respect to the accompanying pulmonary artery, with a lack of tapering of the bronchi |
| Linear density | Focal or multifocal subsegmental atelectasis or fibrosis showing linear configuration, almost always extending to the pleura |
